# Another Dose about the Dose

**Published:** 1890-12

**Authors:** 


### ANOTHER DOSE ABOUT THE DOSE.

A young physician in my old home, the Excelsior State, after giving me full credit for my homœopathic therapeutics, pointedly asks: "Why did not Dr. Lilienthal indicate to the reader the choice of potency for the different remedies used in different diseases? Dear doctor, would it not be an additional legacy to the homœopathic profession, and one which the profession much needs, if you would go through the book once more and indicate to your readers your choice of potency, at least where you have had personal experience in the prescribing of the remedy?"

The answer to my esteemed correspondent was that he asks an impossibility, and that the dosage of drugs was left untrammelled, because, otherwise, it would mislead the reader. When in former times my health allowed me to preside over clinics (and I never felt happier than when surrounded by students), I often told them of the triune factor which determines the dose: 1. the individuality of the patient, with his or her anamnesis. (Hahnemann's psora is not an old man's idle dream); 2. the individuality of the symptom complex, and how closely we have to differentiate between the special symptoms peculiar to the patient and the special symptoms peculiar to the stage of the affection; 3, and finally, the individuality of the drug, so that it may become a remedy. How often did I preach to my hearers to follow Hahnemann's advice in relation to the mental symptoms of the patiént during health and disease, and thus every case becomes a unicum by itself, and no iron-bound rule could be laid down in relation to the dose and when to repeat it.

Just as I was trying to answer my young friend, I received No. 3 of the *Allgemeine Homœopathische Zeitung*, and that dear old man, Dr. Kunkel, of Kiel, answers that question to the point. In relating some of his cases he adds in one, where he prescribed repeated doses of Calcarea-carbonica$^{30}$, twice a day a drop; perhaps less frequent doses might have sufficed, but my experience taught me that where we have to deal with composite morbid products, a frequent repetition of the drug gives better results. The question often comes to my mind, whether the method of our "scientific" opponents, who, on a solitary symptom of a constitutional affection, try to remove it by surgery, is more scientific than our method, which removes all symptoms, and thus, also, the one more prominent. Jahr gives in such cases one dose and expects in all cases a cure, providing he found the simillimum. Perhaps he might have succeeded in one case and then tries to formulate a law upon this single case. In another case of glaucoma, where an operation was contra-indicated and which he greatly relieved by Sepia 2d and 200th, followed by Phosphorus$^{30}$, every seventh evening a powder (a favorite dosage of the doctor, to give one dose once a week), Kunkel adds to the history of the case the simultaneous application of a low potency with a high one in cases where one intends next to its local action upon a diseased organ or upon a chronic product of the disease also to produce a general action; this procedure, recommended by different physicians, remains still *sub judice* (S. L. acknowledges belonging among these "different" physicians, and his vote is in its favor, or it is an alternation based on the symptoms in the provings).

One case more of the old man, on account of homœopathic prescription *versus* gynæcological abuse of surgery. A young woman complained for years of different troubles; of colicky pains before and after menstruation, now constant; the pains are lancinating, from outside inwards, especially when in motion; when sitting down suddenly, stitches in anus. In the morning when rising occipital headache, accompanied by a paretic sensation. She feels best when in the fresh air, but she must not remain out too long. She is irritable and changeable in her moods. Cannot lie so well on left side, worse in windy weather. Collum and corpus uteri enlarged, hard, nearly immovable, painful on pressure. Platina$^{30}$, every seventh evening; one dose restored her health and she thus escaped all gynæcological local treatment.

So far Dr. Kunkel, and my esteemed correspondent might well ask why this old and venerable practitioner gives all his remedies in chronic cases always in the same manner, "every seventh evening one dose"? Is it a mere habit, for it cannot be based on an iron-bound rule. Kunkel is fond of the thirtieth potency, and so was Hahnemann; Bœnninghausen and Dunham prescribed their own hand-made two-hundreth potencies; Kane relies on the five hundreth, one of our Philadelphia masters acknowledges that if he were confined to one potency, he would prefer the sixth to all others; our microscopists deny all medicinal power to anything above the twelfth, because they cannot see the drug; while an extreme high potency may work and does work and cure in spite of all incredulity heaped upon these dynamic factors.

Who will come to the rescue of our younger colleagues, who seek for information and for instruction? Do not give them a stone for bread! Do not say, You must work out your own solution! It is not the first time that the request has been made, but I do not see my way clear how to comply with it. The microscopist and specialist cannot answer it satisfactorily, one must be convinced of the healing power inherent in the dose of the drug, selected with the utmost care, or else it will fail to do good. Let us hear from men who are close prescribers and the younger generation will bless them for it. Amen, Selah!

